# Abnormal cholesterol–cholesteryl ester metabolism impairs mouse oocyte quality during ovarian aging

**DOI:** 10.1186/s11658-025-00811-w

**Published:** 2025-11-24

**Authors:** Sainan Zhang, Bichun Guo, Junshun Fang, Shanshan Wang, Yicen Liu, Die Wu, Nannan Kang, Yang Zhang, Xin Zhen, Guijun Yan, Lijun Ding, Haixiang Sun, Chuanming Liu

**Affiliations:** 1 Center for Reproductive Medicine and Obstetrics and Gynecology, Nanjing Drum Tower Hospital Clinical College of Nanjing Medical University, Nanjing, China; 2 State Key Laboratory of Reproductive Medicine and Offspring Health, Nanjing Medical University, Nanjing, China; 3 Center for Reproductive Medicine and Obstetrics and Gynecology, Nanjing Drum Tower Hospital, Affiliated Hospital of Medical School, Nanjing University, Nanjing, China; 4 Center for Reproductive Medicine and Obstetrics and Gynecology, Joint Institute of Nanjing Drum Tower Hospital for Life and Health, College of Life Science, Nanjing Normal University, Nanjing, China; 5 Center for Molecular Reproductive Medicine, Nanjing University, Nanjing, China; 6 State Key Laboratory of Analytic Chemistry for Life Science, Nanjing University, Nanjing, China; 7 Clinical Center for Stem Cell Research, Nanjing Drum Tower Hospital, Nanjing University, Nanjing, China

**Keywords:** Oocyte maturation, Cholesterol–cholesteryl ester metabolism, ACAT1, Mitophagy, Mitochondrial function, Oocyte aging

## Abstract

**Background:**

Ovarian aging-induced decline in oocyte quality has been a main issue in women of advanced maternal age. However, the potential mechanism remains elusive, and there are no effective strategies to ameliorate aged oocyte quality. The lipid metabolism of oocytes has drawn great attention, but the intrinsic regulation of oocyte quality by metabolites, metabolic enzymes, and intracellular mediators is less well-characterized.

**Methods:**

Targeted lipidomics was employed to detect the neutral lipids in oocytes during maturation. We used 4,4-difluoro-1,3,5,7,8-pentamethyl-4-bora-3*a*,4*a*-diaza-*s*-indacene (BODIPY 493/503) and Filipin to stain cholesteryl ester and free cholesterol, respectively. The Cholesterol/Cholesteryl Ester Quantification Assay kit was used further to quantify cholesterol-related metabolites. Western blotting was performed to evaluate acyl-coenzyme A: cholesterol acyltransferase 1/2 (ACAT1/2) expression. Immunofluorescence and quantitative real-time polymerase chain reaction (qRT-PCR) were conducted to validate the knockdown efficiency of ACAT1. Avasimibe treatment and ACAT1 small interfering RNA (siRNA) microinjection were performed to investigate the effect of impaired cholesterol–cholesteryl ester metabolism on oocyte quality. Single-oocyte RNA sequencing was conducted to explore the mechanism. Mitochondrial membrane potential (MMP), adenosine triphosphate (ATP) production, reactive oxygen species (ROS), and mitochondrial autophagosomes were detected to evaluate mitochondrial function and mitophagy.

**Results:**

There is a profound increase in the conversion of cholesterol to cholesteryl ester in oocytes during maturation, which depends on ACAT1. Conversely, disturbing the homeostasis of cholesterol–cholesteryl ester metabolism by manipulating ACAT1 impairs oocyte quality, primarily manifested as decreased polar body extrusion (PBE), increased meiotic defects, and abnormal early embryonic development. Mechanistically, the impaired conversion of cholesterol to cholesteryl ester reduces oocyte mitophagy, leading to mitochondrial dysfunction, including reduced MMP and ATP production, and excessive accumulation of ROS. Notably, we also reveal that this metabolic homeostasis is impaired in aged oocytes, accompanied by decreased ACAT1 levels. Moreover, cholesteryl ester supplementation via cholesterol conjugated to methyl-β-cyclodextrin (CCM) can effectively ameliorate aged oocyte quality by enhancing mitophagy.

**Conclusions:**

This study reveals the mechanism by which cholesterol–cholesteryl ester metabolism regulates oocyte quality and thus participates in the process of oocyte aging by influencing mitophagy and mitochondrial function.

**Graphical Abstract:**

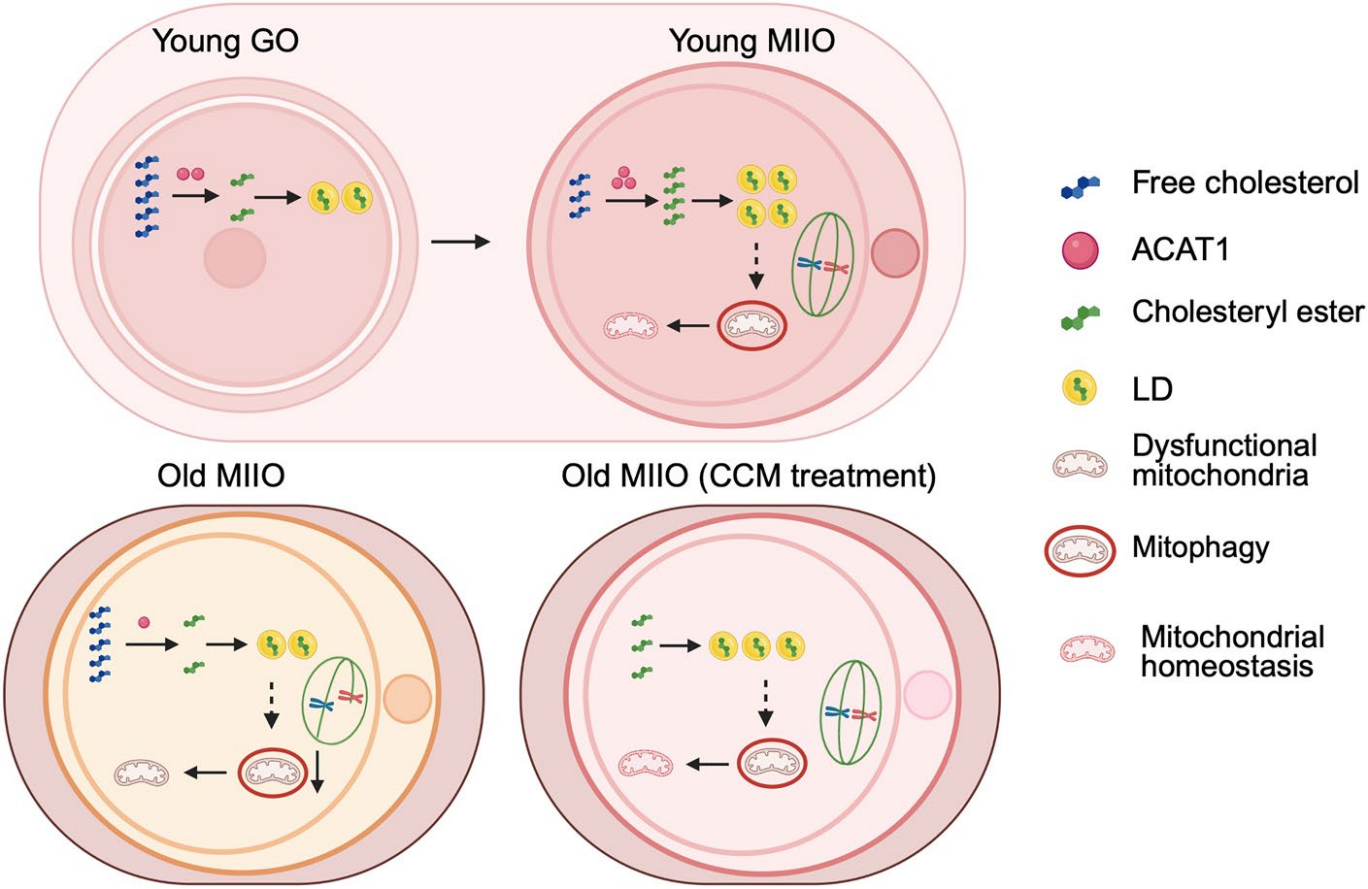

**Supplementary Information:**

The online version contains supplementary material available at 10.1186/s11658-025-00811-w.

## Background

The continued trend of delayed childbearing has become increasingly common in the past decades worldwide, with ensuing medical problems attributed primarily to maternal aging [1]. With increasing age, the fertility of women decreases dramatically, particularly after 35 years old [2]. In addition, advanced maternal age is associated with an increased risk of a wide range of pregnancy complications, including miscarriages, chromosomal abnormalities, and other adverse outcomes [3–5]. As the primary female reproductive organ, the ovary shows earlier aging than other organs [6, 7]. In addition to the decrease in oocyte number, the decline in oocyte quality caused by ovarian aging primarily explains the aforementioned issues [8]. Ovarian aging-induced oocyte quality decrease is closely related to oocyte meiotic maturation abnormalities, ultimately leading to increased oocyte aneuploidy [9, 10]. A great deal of work has been devoted to exploring the potential mechanism of aging-related decline in oocyte quality. Recently, the metabolic regulation of oocyte aging has drawn much attention.

Many key metabolic factors undergo changes and thus impact oocyte quality during the ovarian aging process. The decrease in nicotinamide adenine dinucleotide levels as ovarian aging progresses damages oocyte quality [11]. Our previous work reported that decreased granulosa cell mevalonate pathway contributed to aged oocyte meiotic defects and aneuploidy [12]. Moreover, correcting the metabolic imbalance has been demonstrated effectively to restore aged oocyte quality. The polyamine metabolite spermidine can rejuvenate oocyte quality during female reproductive aging [13]. Mitochondria also play an indispensable role in the cellular metabolism and energy homeostasis of oocytes [14, 15]. In summary, these findings emphasize that a well-balanced metabolism is essential for maintaining oocyte quality.

Previous studies in mammals have underscored that lipid metabolism provides sufficient energy for morphological and cellular events [16–18]. Lipids are also essential signaling molecules involved in various biological cascades of oocyte growth, maturation, and competence acquisition [19]. It is well known that oocytes contain lipid droplets (LDs), which are ubiquitous cellular organelles responsible for storing neutral lipids, including triacylglycerol (TAG) and cholesteryl ester [20, 21]. A previous study reported the role of TAG in oocyte development and maturation [22]. However, the role of cholesteryl ester in the oocyte is unknown. Cholesteryl ester is formed by cholesterol esterification to prevent excess accumulation of free cholesterol. Cholesterol is traditionally considered essential for female fertility, mainly on the basis of its role in the steroid synthesis of the ovary. In addition, it is reported that proper cholesterol is associated with oocyte maturation while excessive cholesterol induces abnormal premature activation [23–26]. These findings highlight the potential importance of cholesterol–cholesteryl ester homeostasis. Whether the homeostasis of cholesterol–cholesteryl ester metabolism is involved in regulating oocyte quality and ovarian aging, however, remains elusive.

In the current study, we evaluate the change of neutral lipids in oocytes during maturation and show a profound increase in the conversion of cholesterol to cholesteryl ester during oocyte maturation. Moreover, this metabolic conversion depends on a pace-limiting enzyme, acyl-coenzyme A: cholesterol acyltransferase 1 (ACAT1). Conversely, disrupting this metabolism by manipulating ACAT1 leads to an impairment of oocyte quality, including decreased polar body extrusion (PBE), increased meiotic defects, and abnormal early embryonic development. Mechanistically, impaired cholesterol–cholesteryl ester homeostasis downregulates oocyte mitophagy, resulting in decreased mitochondrial membrane potential (MMP) and adenosine triphosphate (ATP) production, and excess accumulation of reactive oxygen species (ROS). Remarkably, we also confirm imbalanced cholesterol–cholesteryl ester metabolism in aged oocytes, accompanied by significantly decreased ACAT1 levels, and correcting this metabolic imbalance can effectively rescue aged oocyte quality via improving mitophagy.

## Materials and methods

### Animals

Female C57BL/6 mice were purchased from SPF Biotechnology Company (Beijing, China). Young and aged mice were defined on the basis of age of 6 weeks and 10 months, respectively. All mice were housed in the animal laboratory center of Nanjing Drum Tower Hospital under specific pathogen-free conditions, providing a standard 12-h light–dark cycle, proper temperature and humidity, and adequate water and food. Animal care and experimental procedures were performed following the guidelines of the experimental animal management committee (Jiangsu, China). The Experimental Animal and Welfare Ethics Committee of Nanjing Drum Tower Hospital approved all animal experimentation procedures (approval no. 2023AE01085, approved 10 October 2023).

### Oocyte collection and lipidomics analysis

**Fig. 1:**
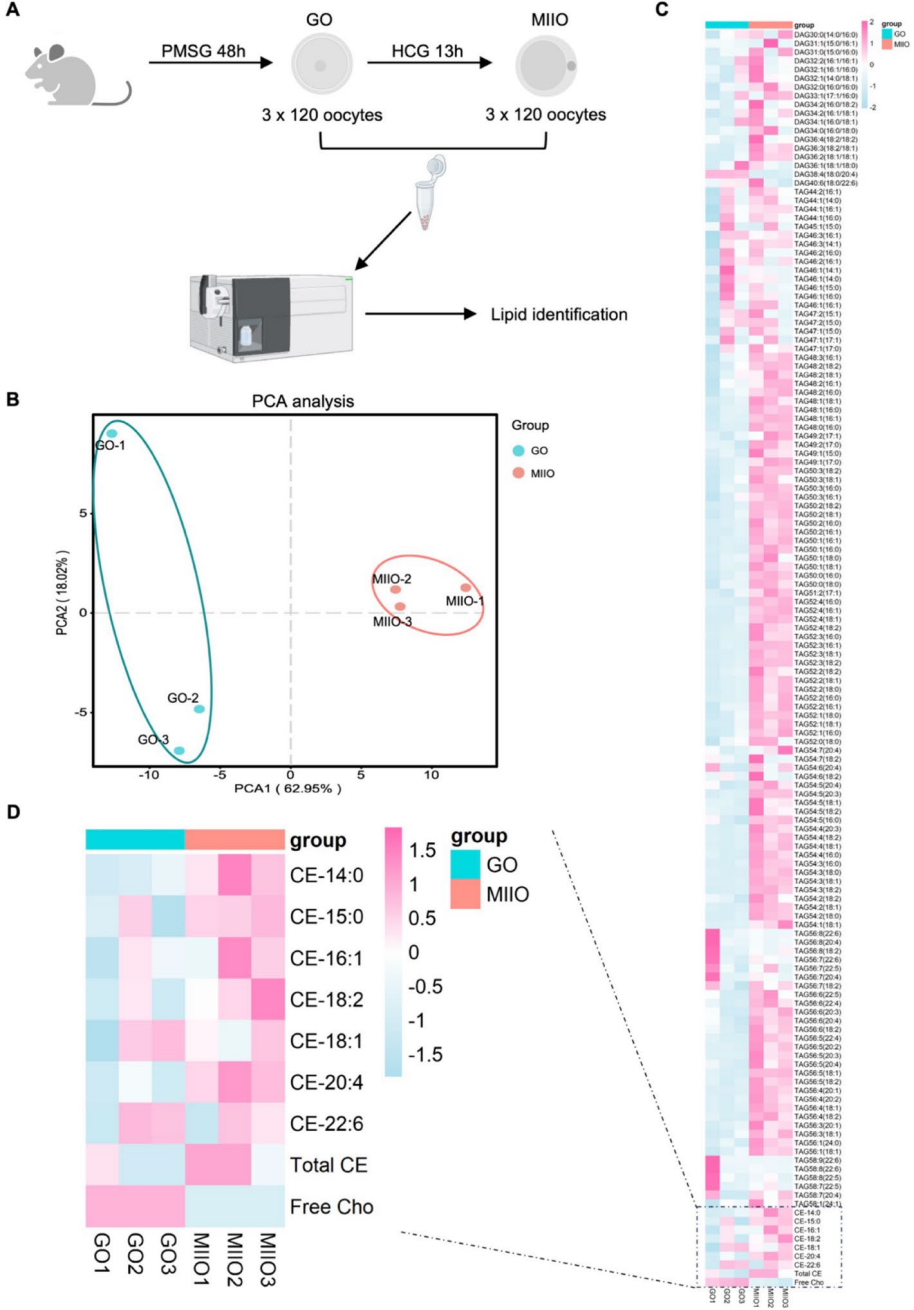
Lipidomics analysis of the mouse oocyte during maturation. **A** Schematic workflow of in vivo isolation of 360 GOs and 360 MIIOs for lipidomics detection. *GO* germinal vesicle oocyte, *MIIO* metaphase II oocyte, *PMSG* pregnant mare serum gonadotropin, *HCG* human chorionic gonadotropin. **B** PCA of lipidomics metabolic profiling in the GO and MIIO groups. *PCA* principal component analysis. **C** Heatmap showing detected lipids in the GO and MIIO groups. The color scale on the right represents relative expression levels; red represents high levels, and blue represents low levels. *DAG* diacylglycerol, *TAG* triacylglycerol, *Cho* cholesterol, *CE* cholesteryl ester. **D** Heatmap showing free cholesterol and cholesteryl ester abundances in the GO and MIIO groups

The processes of oocyte collection and lipidomics analysis were executed as shown in Fig. 1A. In brief, to maximize the acquisition of the denuded germinal vesicle oocytes (GOs), female 6-week-old C57BL/6 mice were given 10 IU of pregnant mare serum gonadotropin (PMSG) (Sansheng Pharmaceuticals, Ningbo, China) through intraperitoneal injection. After 48 h, the mice were sacrificed, and the ovaries were separated and transferred to an M2 medium (Sigma-Aldrich, M7167, St. Louis, MO, USA). After removing the peri-ovarian adipose tissue under a Nikon SMZ1270 stereomicroscope, the GOs were obtained by cutting the ovaries with a blade. For the metaphase II oocytes (MIIOs), an additional 10 IU of human chorionic gonadotropin (HCG) (Sansheng Pharmaceuticals, Ningbo, China) was administered via intraperitoneal injection 48 h after 10 IU PMSG administration. The cumulus–oocyte complexes (COCs) were immediately harvested from the oviductal ampulla 13 h later and were digested in hyaluronidase medium (Sigma-Aldrich, 9001-54-1, St. Louis, MO, USA) to remove the surrounding granulosa cells. Finally, a total of 360 GOs and 360 MIIOs were collected for each group with three repeats.

The lipids were extracted using a modified version of Bligh and Dyer’s method as described previously [27]. In brief, the oocytes were homogenized in 750 µL of chloroform:methanol:MilliQ H_2_O (3:6:1). Then, the homogenate was incubated with an optoelectronically controlled small IKA shaker at 1500 rpm for 1 h at 4℃. Next, 350 µL of deionized water and 250 µL of chloroform were added to induce phase separation. Next, the samples were centrifuged at 12,000 rpm for 5 min at room temperature, and the lower, organic phase containing lipids was collected into a clean tube. Lipid extraction was repeated once by adding 450 µL of chloroform to the remaining cells in the aqueous phase, and the lipid extracts were pooled into a single tube and dried in the SpeedVac under H_2_O mode. The samples were stored at −80 ℃ until further analysis. The upper aqueous phase and cell pellet were dried in a SpeedVac under H_2_O mode. Glycerol lipids, including diacylglycerol (DAG) and TAG, were quantified using a modified version of reversed-phase high-performance liquid chromatography (HPLC) with multiple reaction monitoring [28]. Neutral lipids separation was achieved on a Phenomenex Kinetex-C18 column (i.d. 4.6 × 100 mm, 2.6 µm) via an isocratic mobile phase containing chloroform:methanol:0.1 M ammonium acetate 100:100:4 at a flow rate of 300 µL for 10 min. Levels of short-, medium-, and long-chain TAG were calculated by reference to spiked internal standards of TAG (14:0) 3-d5, TAG (16:0) 3-d5, and TAG (18:0) 3-d5 obtained from CDN isotopes, respectively. Quantification of DAG was done using d5-DAG17:0/17:0 and d5-DAG18:1/18:1 as internal standards (Avanti Polar Lipids). Analysis of free cholesterol and cholesteryl ester was made under atmospheric pressure chemical ionization mode on a Jasper HPLC coupled to Sciex 4500 MD as per our previous description, using d6-cholesterol and d6-C18:0 cholesteryl ester (CDN isotopes) as internal standards [29]. Analysis and visualization for lipidomics data are described as follows: Principal component analysis (PCA) was conducted on the centered and scaled values using the R package “FactoMineR” (2.12). Hierarchical clustering with complete linkage was visualized using the R package “ComplexHeatmap” (2.10). All detected metabolites are shown in the heatmaps, represented by colors ranging from blue to red according to their expression abundance from low to high.

### Denuded oocyte in vitro maturation (IVM) and treatment

The 6-week-old C57BL/6 female mice were given 10 IU PMSG for 48 h through intraperitoneal injection to maximally obtain denuded GOs as described above. Then, the GOs were placed in M16 medium (Sigma-Aldrich, M7292, St. Louis, MO, USA) covered with liquid paraffin oil (Aibei Biotechnology, M2470, Nanjing, China) in a 5% CO_2_ incubator for IVM. The concentration of avasimibe (MCE, HY-13215, Shanghai, China) was 20 μM. The rate of germinal vesicle breakdown (GVBD) and PBE was analyzed after culturing in IVM for 4 h and 14 h, respectively. Then, the MIIOs were retrieved for further experiments.

### COC acquisition and treatment

The 6-week-old and 10-month-old C57BL/6 female mice were given 10 IU PMSG for 48 h through intraperitoneal injection, and the ovaries were obtained as described above. COCs were released from the ovarian antral follicles using a disposable syringe with a 20-gauge needle under a stereomicroscope. They were then incubated in M16 medium covered with liquid paraffin oil at 37 ℃ with 5% CO_2_. Cholesteryl ester addition was conducted with the cholesterol (Sigma-Aldrich, C8667, St. Louis, MO, USA) conjugated to methyl-β-cyclodextrin (Sigma-Aldrich, C4555, St. Louis, MO, USA) at a molar ratio of 1:20 (cholesterol conjugated to methyl-β-cyclodextrin, CCM) for a final concentration of 20 μM, on the basis of a previous report [30]. After culturing for 4 h and 14 h, they were transferred into hyaluronidase to isolate oocytes for evaluating the GVBD and PBE rates. Then, the MIIOs were retrieved for further experiments.

### IVM, in vitro fertilization (IVF), and embryo culture

The 6-week-old and 10-month-old C57BL/6 female mice were intraperitoneally injected with 10 IU PMSG and sacrificed 48 h later. The COCs or denuded oocytes (DOs) were isolated as described above, and they were cultured in minimum essential medium α (MEMα; Gibco, 32561037, Waltham, MA, USA) maturation medium covered with liquid paraffin oil in an incubator at 37℃ with 5% CO_2_. The MEMα maturation medium contained 10% fetal bovine serum (FBS; Gibco, 10270106, Waltham, MA, USA), 10 ng/mL epidermal growth factor (EGF; Gibco, 53003-018, Waltham, MA, USA), and 1.5 IU/mL HCG. For IVF, sperm were released from the epididymides of 12-week-old C57BL/6 male mice by puncture and were subsequently capacitated for 1 h in human tubal fluid medium (Sigma-Aldrich, MR-070, St. Louis, MO, USA). The DOs or COCs after IVM for 14 h were added to be fertilized by addition of capacitated sperm for 6 h in a 37 °C incubator with 5% CO_2_. The fertilized oocytes were subsequently transferred to potassium simplex optimized medium (KSOM; Sigma-Aldrich, MR-106-D, St. Louis, MO, USA) for subsequent culture. The two-cell embryo rates were subsequently calculated.

### BODIPY 493/503 staining

BODIPY 493/503 staining of oocytes was conducted following the instructions. In brief, oocytes were incubated with BODIPY 493/503 (Beyotime, C2053S, Shanghai, China) diluted in the buffer at room temperature for 30 min, followed by washing three times with M2 medium. The distribution of neutral lipids was observed and recorded by an inverted microscope (Leica, DMI3000B).

### Filipin staining

Filipin staining of oocytes was executed on the basis of the manufacturer’s description. Simply, oocytes were first fixed in 4% paraformaldehyde (PFA; Beyotime, P0099, Shanghai, China) for 10 min and then washed with phosphate-buffered saline (PBS; Gibco, C10010500CP, Carlsbad, CA, USA) three times. Then incubation of oocytes was performed with 0.05 mg/mL Filipin complex (MCE, HY-N6716, Shanghai, China) diluted by PBS at room temperature for 30 min. After washing again three times with PBS, the free cholesterol was detected by an inverted microscope.

### Cholesterol and cholesteryl ester assay

Cholesterol and cholesteryl ester were detected using the Cholesterol/Cholesteryl Ester Quantification Assay kit (Abcam, ab65359, Cambridge, MA, USA), following the instructions. In brief, 50 oocytes were collected for each group, washed with PBS, and then transferred to tubes. Lipid extract was done by resuspending the oocyte sample in 200 μL of chloroform:isopropanol:NP-40 (7:11:0.1) in a microhomogenizer. Subsequently, the extract was spun for 10 min at 15,000 rpm in a centrifuge before transferring all of the liquid (organic phase), avoiding the pellet, to a new tube, then air-dried at 50℃ to remove chloroform and subject to vacuum for 30 min to remove trace organic solvent. Finally, dried lipids were dissolved with 200 μL of assay buffer II/assay buffer for the next step. For the detection well, 50 μL of standard dilution was added, while the total cholesterol sample well and the free cholesterol sample well were given 50 μL of sample mixture. Total cholesterol reaction mixture was prepared, including 45.6 μL of assay buffer II/cholesterol assay, 0.4 μL of cholesterol probe, 2 μL of enzyme mix I/cholesterol enzyme mix, and 2 μL of cholesterol esterase, while the free cholesterol reaction mixture contained 47.6 μL of assay buffer II/cholesterol assay, 0.4 μL of cholesterol probe, and 2 μL of enzyme mix I/cholesterol enzyme mix, omitting the cholesterol esterase. Following this, 50 μL of the total cholesterol reaction mixture was added to both the standard well and the total cholesterol sample well, while the free cholesterol sample received 50 μL of the free cholesterol reaction mixture. The setup was then incubated in the dark at 37 ℃ for 60 min. Finally, fluorescence measurements were conducted using the automated Tecan Spark multimode microplate reader (Tecan, Switzerland), setting the excitation and emission wavelength at 535 and 587 nm, respectively.

### Immunofluorescence

Oocytes were fixed in 4% PFA for 30 min, then permeabilized with 0.5% Triton X‐100 (Sigma-Aldrich, T9284, St. Louis, MO, USA) for 20 min. Following three washes with 1% bovine serum albumin (BSA; Sigma-Aldrich, SRE0096, St. Louis, MO, USA), the oocytes were blocked with 3% BSA for 1 h at room temperature. The primary antibodies were incubated overnight at 4 °C with mouse anti-α-tubulin (Sigma-Aldrich, cat. no. F2168, lot no. 0000167896, St. Louis, MO, USA, 1:700) or rabbit anti‐ACAT1 (Abcam, cat. no. ab307597, lot no. 1036351-7, Cambridge, MA, USA, 1:200). Following three washes with phosphate buffered saline with Tween-20 (PBST), oocytes were treated with goat anti-rabbit IgG (H+L) cross-adsorbed secondary antibody, Alexa Fluor™ 594 (Invitrogen, cat. no. A-11012, lot no. 2616076, Waltham, MA, USA, 1:200) for 1 h at room temperature. The samples were then washed three times with PBST for 5 min. Finally, the 4′,6-diamidino-2-phenylindole (DAPI)-containing anti-fluorescence quencher (Invitrogen, cat. no. S36968, lot no. 2359200, Waltham, MA, USA) was applied to glass slides, onto which the oocytes were then placed before covering with a coverslip. The mounted oocytes were observed using a Leica microscope (DM3000 LED). For staining α-tubulin, the step of secondary antibodies should be omitted.

### Small interfering RNA (siRNA) transfection

The design and synthesis of ACAT1 siRNA were carried out by General Biol (Chuzhou, China). Mouse neuroblastoma cells (Neuro-2a) were cultured with Dulbecco’s modified Eagle’s medium (DMEM) medium (Gibco, C11995500BT, Waltham, MA, USA), added with 10% FBS and 1% penicillin–streptomycin (Gibco, 15140122, Waltham, MA, USA) in a humidified environment with 5% CO_2_ at 37 °C. When the cells reached 70% confluence, siRNA transfection was done as the following assay. Firstly, the 5 μg of ACAT1 siRNA and 5 μL of Lipofectamine 3000 reagent (Invitrogen, L3000001, Carlsbad, CA, USA) were diluted with 250 μL of OptiMEM (Gibco, 11058021, Grand Island, NY, USA), respectively. The ACAT1 siRNA mixture was then gently added to the Lipofectamine 3000 system for incubation for 20 min at room temperature. The compound was subsequently added to the cells for transfection for 8 h before the medium was refreshed. The negative control transfection was performed in the same way. All the above treatments lasted for 48 h before arrest. The sequences of ACAT1 siRNA and negative control are listed in Table 1.

**Table 1:** Primer sequences

Primer		Sequence (5′–3′)
ACAT1	Forward	TGAGAGCACCTCCAGAACAAGG
	Reverse	GGACGAATAGGATGAGGAGTGC
18S	Forward	ATGGCCGTTCTTAGTTGGTG
	Reverse	CGGACATCTAAGGGCATCAC
ACAT1 siRNA	Sense	AGAAGAUGUCACUAAGAAATT
	Antisense	UUUCUUAGUGACAUCUUCUTT
Negative control siRNA	Sense	UUCUCCGAACGUGUCACGUTT
	Antisense	ACGUGACACGUUCGGAGAATT

### Oocytes microinjection

After confirming the efficiency of ACAT1 siRNA knockdown, ACAT1 siRNA was diluted with RNase-free water to achieve a 20 μM stock solution. GOs were collected as described above. About 5 pL of ACAT1 siRNA was injected into the oocyte using a FemtoJet microinjector (Eppendorf) with an equal volume of negative control. After injection, the oocytes were cultured in an M16 medium supplied with 5 μM milrinone (Sigma-Aldrich, PHR2515, St. Louis, MO, USA) for 18 h. Then, oocytes were washed five times in a fresh M2 medium to remove milrinone and transferred to a fresh M16 medium for IVM.

### Quantitative real-time PCR (qRT-PCR)

Amplification of oocyte mRNA was executed using a Single Cell Sequence-Specific Amplification Kit (Vazyme, P621-01, Nanjing, China) following the manufacturer’s protocol. In brief, oocytes were first collected in the RNase-free tubes, individually. An assay pool with a final concentration of 0.1 μM for each primer was then prepared. Finally, a 5 μL reaction mixture was constituted by 2.5 μL 2× reaction Mix, 0.5 μL assay pool, 0.1 μL RT/Taq enzyme, 0.9 μL RNase-free water, and 1 μL oocyte sample for PCR reaction. For qRT-PCR, ChamQ Universal SYBR qPCR Master Mix (Vazyme, Q711-02, Nanjing, China) was used based on the instrument. The reaction mixture comprised 10 μL of SYBR-Green Mixture, 0.5 μL of forward primer, 0.5 μL of reverse primer, 2 μL of complementary DNA (cDNA), and 7 μL of ddH_2_O. Relative mRNA expression was normalized to 18S and calculated using the 2^−ΔΔCt^ method. The mouse primer sequences are presented in Table 1.

### Protein isolation and western blotting

Cells were lysed at 4 °C for 30 min using radioimmunoprecipitation assay (NCM biotech, WB3100, Suzhou, China) lysis buffer with protease inhibitor cocktail (Roche, 4693132001, Basel, Switzerland). The lysis was then centrifuged at 4 °C and 12,000 rpm for 15 min to collect supernatant protein. Quantification was done with a bicinchoninic acid (BCA) kit (Thermo Fisher Scientific, Waltham, MA, USA, 23227) following the manufacturer’s instructions. For oocyte protein collection, 70 oocytes in each group were lysed with a 20 μL lysis buffer for 30 min and then heated at 95℃ for 10 min. Western blotting was performed as previously described [31]. In brief, proteins were separated by sodium dodecyl sulfate (SDS)-polyacrylamide gel electrophoresis (PAGE) on 10% gels and transferred to polyvinylidene fluoride membranes (Millipore, 03010040001, Billerica, MA, USA). The membranes were blocked with 5% nonfat milk for 1 h at room temperature and then incubated overnight at 4 °C with primary antibodies: rabbit anti‐ACAT1 (Abcam, cat. no. ab307597, lot no. 1036351-7, Cambridge, MA, USA, 1:1000), rabbit anti‐ACAT2 antibody (Proteintech, cat. no. 21852-1-AP, lot no. 00088056, Wuhan, China, 1:1000), and rabbit polyclonal anti-β-actin (Abmart, cat. no. P30002M, lot no. 374170, Shanghai, China, 1:2000). After three washes with PBST, the membranes were incubated with horseradish peroxidase (HRP)-conjugated secondary antibodies (Zsbio, cat. no. ZB-2301, lot no. 247860317, Beijing, China, 1:10,000) and for 1 h at room temperature. After washing again, the membrane was visualized using enhanced chemiluminescence reagents (Millipore, WBKLS0500, Billerica, MA, USA).

### Single-oocyte RNA sequencing and analysis

Single-oocyte RNA sequencing was performed as our previous assay [12]. Simply, total RNA from the oocytes was reverse-transcribed into cDNA using the Discover-sc™ WTA Kit V2 (Vazyme, N711, Nanjing, China) following the manufacturer’s guidelines. Subsequently, libraries were constructed utilizing the TruePrep™ DNA Library Prep Kit V2 for Illumina (Vazyme, TD503, Nanjing, China). Library sequencing and subsequent analyses were conducted on the Illumina HiSeq X platform (Majorbio). RNA sequencing data were assessed for the overall clustering profile using the “psych” package in R. PCA was performed using the FactoMineR (2.12) and factoextra (1.3) packages in RStudio. Differentially expressed genes (DEGs) were identified by employing the DESeq (1.46.0) package. Kyoto Encyclopedia of Genes and Genomes (KEGG) analysis was achieved with the database for Annotation, Visualization, and Integrated Discovery ClusterProfiler (4.14.6).

### Mitochondrial autophagosome detection

Detection of mitochondrial autophagosomes was performed using a Mitophagy detection kit (Dojindo, MD01, Kumamoto, Japan). In brief, oocytes were incubated with a 100 nM mitophagy dye at 37 °C for 30 min. After washing three times, an inverted fluorescence microscope was used to capture the images.

### MMP detection

MMP detection of oocytes was conducted according to the Enhanced Mitochondrial Membrane Potential Assay Kit (Beyotime, C2003S, Shanghai, China). In simple, oocytes were incubated at 37 ℃ for 20 min with the 1 × JC-1 diluted by the assay buffer. After washing three times with the assay buffer, the inverted fluorescence microscope was used to observe the JC-1 aggregates and monomers.

### ROS detection assay

MitoSOX Green (Invitrogen, M36005, Carlsbad, CA, USA) was used to examine the ROS levels in oocytes. Firstly, oocytes were incubated at 37 ℃ for 15 min with MitoSOX Green at a concentration of 1 μM. After washing, inverted fluorescence microscopy was used to detect the ROS levels.

### ATP detection

Mitochondrial ATP content was examined according to the manufacturer’s instructions (Beyotime, S0027, Shanghai, China). Firstly, standard solutions of ATP were prepared by diluting the ATP standard solution with ATP detection lysis buffer to generate a suitable concentration gradient of 0.01, 0.03, 0.1, 0.3, 1, 3, and 10 μM. For sample preparation, ten oocytes in each group were obtained using the previously described method and placed into 20 μL lysis buffer. After adding 100 μL of ATP detection working solution to the detection well at room temperature for 5 min, a 20 μL sample or standard solution was added to the detection well and mixed quickly using a pipette. Measurement was made with the automated Tecan Spark multimode microplate reader. The ATP content was calculated via a standard curve.

### Statistical analysis

All experiments were independently repeated at least three times, including at least three mice in each group, for each experiment. Data are presented as mean ± standard deviation (SD) and were analyzed by Student’s *t* test. ImageJ (NIH, USA) was employed to analyze images. Statistical analysis and graphing were completed by GraphPad Prism 9.0 Software (San Diego, CA, USA). *P* < 0.05 was considered significant.

## Results

### Lipidomics analysis of the mouse oocyte during maturation

To characterize the alterations of specific lipid metabolites, including DAG, TAG, cholesterol, and cholesteryl ester, during the oocyte maturation process, we conducted targeted lipidomics. The workflow was set up as shown in Fig. 1A. A total of 360 GOs and 360 MIIOs were collected at the indicated times for lipidome detection. PCA results showed that oocyte samples from two different stages were naturally separated into two distinct clusters, which indicated that different oocyte stages exhibit distinct lipidomics signatures (Fig. 1B). A total of 143 lipid metabolites, including 18 types of DAG, 117 types of TAG, 1 type of free cholesterol, and 7 types of cholesteryl ester, were detected (Fig. 1C). Of those, the great majority of DAG and TAG showed markedly increased levels during oocyte maturation. Notably, we found that, compared with the GO group, free cholesterol was remarkably decreased in the MIIO group (Fig. 1D). On the contrary, most cholesteryl ester levels in the MIIO group were significantly increased, especially for CE-14:0, CE-15:0, CE-16:1, CE-18:2, and CE-20:4 (Fig. 1D). To provide a comprehensive overview of cholesteryl ester changes, we further calculated the total cholesteryl ester and found a higher level in the MIIO group than in the GO group (Fig. 1D). Therefore, these findings preliminarily suggest a potential conversion from cholesterol to cholesteryl ester during oocyte maturation.

### Increased conversion of cholesterol to cholesteryl ester during oocyte maturation

**Fig. 2:**
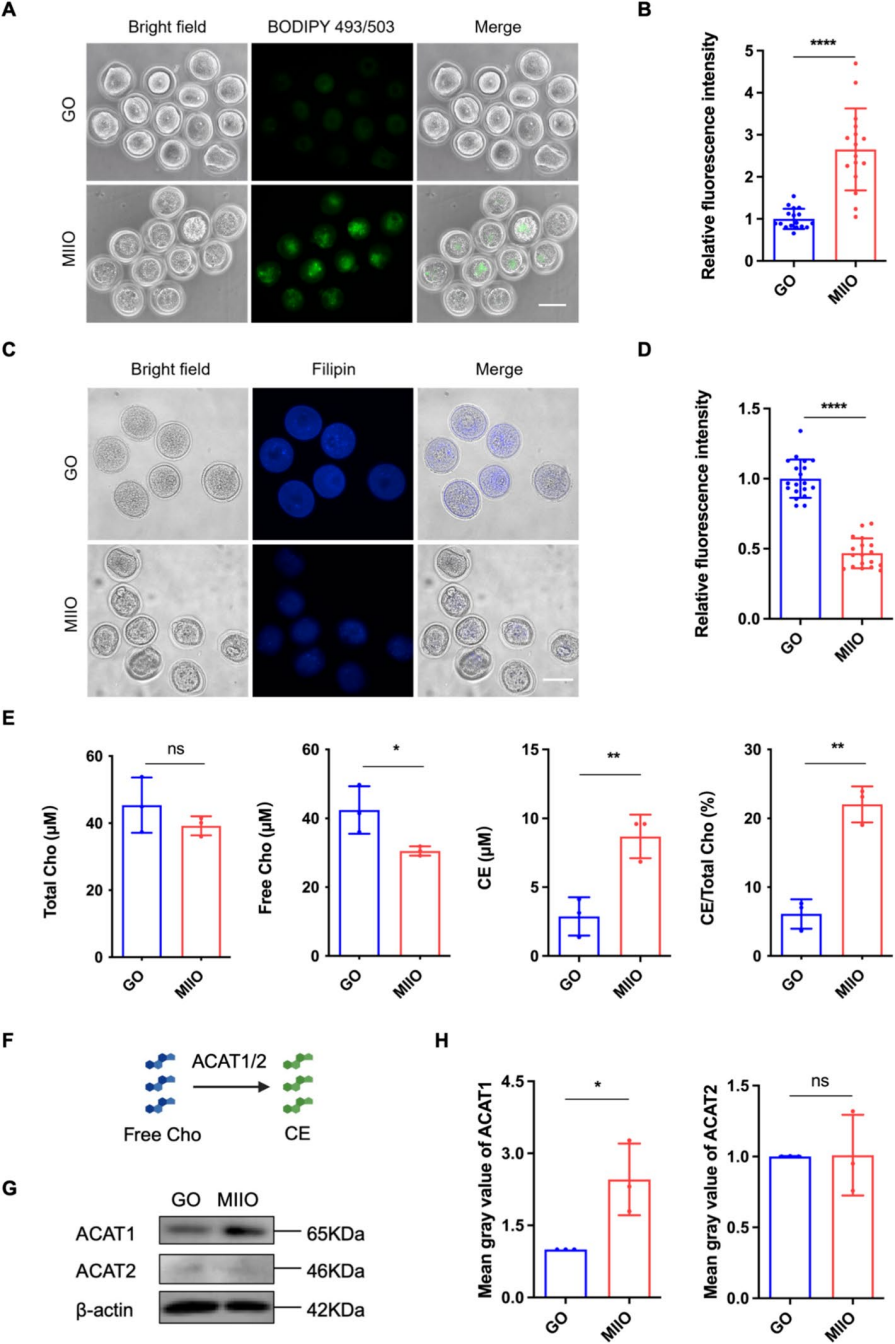
Increased conversion of cholesterol to cholesteryl ester during oocyte maturation. **A** Representative image of BODIPY 493/503 staining in the GO and MIIO groups. *GO* germinal vesicle oocyte, *MIIO* metaphase II oocyte. Scale bar, 100 μm. **B** Relative fluorescence intensity of BODIPY 493/503 staining in the GO (*n* = 17) and MIIO (*n* = 16) groups. **C** Representative images of Filipin staining in the GO and MIIO groups. Scale bar, 100 μm.** D** Relative fluorescence intensity of Filipin staining in the GO (*n* = 19) and MIIO (*n* = 18) groups. **E** Quantification of total cholesterol, free cholesterol, cholesteryl ester, and cholesteryl ester conversion rates in the GO (*n* = 150) and MIIO (*n* = 150) groups. *Cho* cholesterol, *CE* cholesteryl ester. **F** Schematic overview of free cholesterol conversion to cholesteryl ester. *ACAT1/2* acyl-coenzyme A: cholesterol acyltransferase 1/2. **G** Protein expression of ACAT1 and ACAT2 in the GO (*n* = 210) and MIIO (*n* = 210) groups. **H** Mean gray value of ACAT1 and ACAT2 protein expression in the GO and MIIO groups. * *P* < 0.05; ** *P* < 0.01; **** *P* < 0.0001; *ns* not significant

To further validate this metabolic change indicated by the lipidomics analysis, we conducted BODIPY 493/503 and Filipin staining in GOs and MIIOs to detect cholesteryl ester and free cholesterol levels, respectively. Quantification of BODIPY 493/503 suggested a higher level of cholesteryl ester in the MIIO group than in the GO group (Fig. 2A, B). Filipin staining showed that the free cholesterol level in the MIIO group was significantly lower than that in the GO group (Fig. 2C, D). We further quantified the abundance of cholesterol-related metabolites, including total cholesterol, free cholesterol, and cholesteryl ester, with a detection kit. The result showed that there was no significant difference in total cholesterol levels between the GO and MIIO groups (Fig. 2E). However, we found considerably increased cholesteryl ester levels and decreased free cholesterol levels in the MIIO group compared with the GO group, which were consistent with the above lipidomics and staining results (Fig. 2E). Moreover, we also calculated the cholesteryl ester conversion rate and observed a significantly increased ratio of cholesteryl ester/total cholesterol from the GO to MIIO (Fig. 2E).

Cholesterol is esterified to cholesteryl ester via two isoenzymes, acyl-coenzyme A: cholesterol acyltransferase 1/2 (ACAT1/2) in mammals (Fig. 2F). To clarify the crucial enzyme, we assessed the ACAT1 and ACAT2 expression in GOs and MIIOs. The western blotting results showed that there was no difference in the expression of ACAT2, while the ACAT1 level was significantly upregulated in the MIIO group compared with that in the GO group (Fig. 2G, H). Together with the above findings, we depict a drastic increase in cholesterol conversion to cholesteryl ester during oocyte maturation, and this metabolic conversion depends on upregulated ACAT1 expression.

### Disturbance of cholesterol–cholesteryl ester homeostasis interferes with oocyte quality

**Fig. 3:**
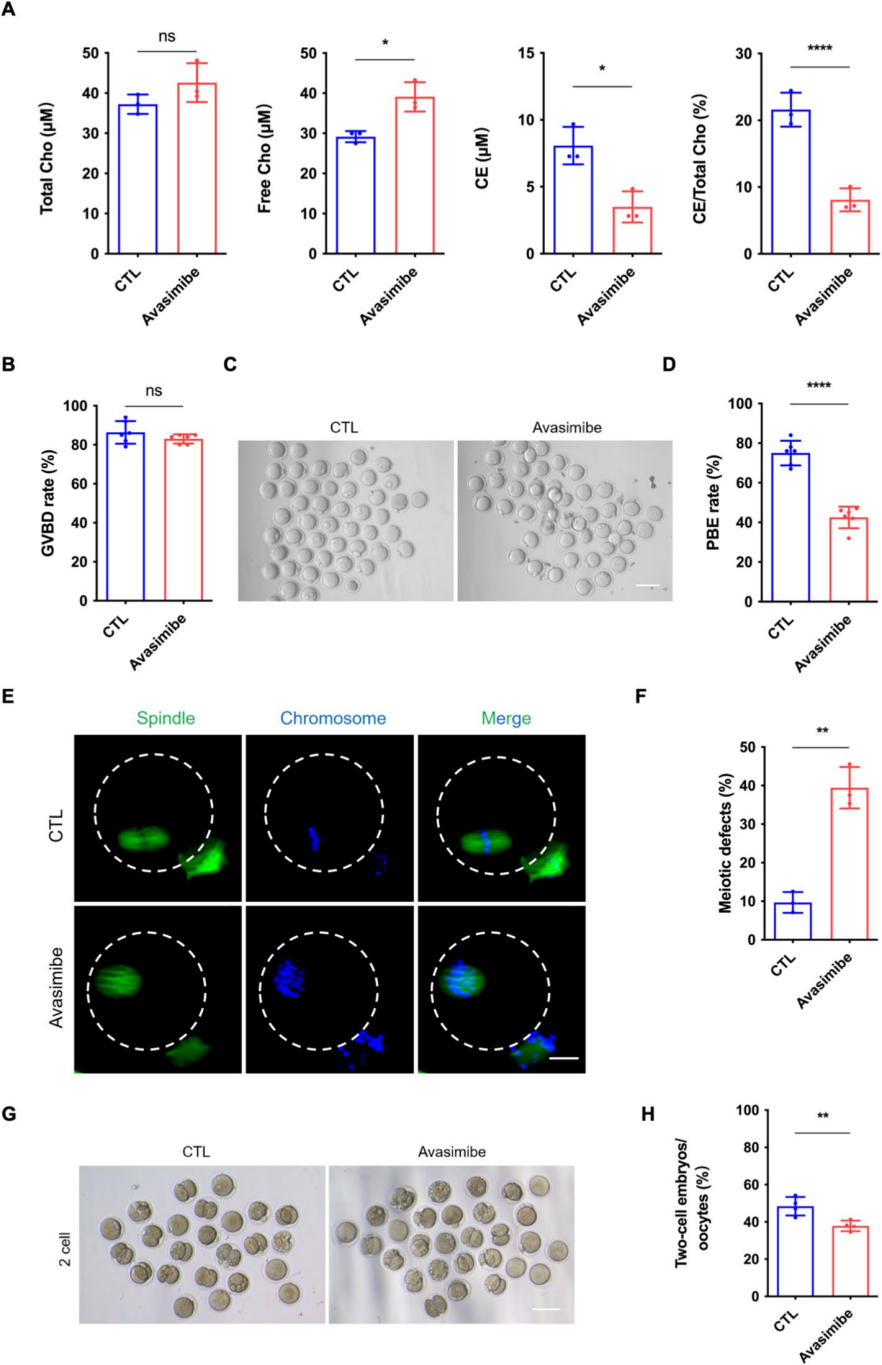
Disturbance of cholesterol–cholesteryl ester homeostasis by avasimibe interferes with oocyte quality. **A** Quantification of total cholesterol, free cholesterol, cholesteryl ester, and cholesteryl ester conversion rates of MIIOs in the CTL (*n* = 150) and avasimibe (*n* = 150) groups. CTL (control) group: oocytes cultured in M16 medium; avasimibe group: oocytes cultured in M16 medium supplemented with 20 μM avasimibe. *MIIOs* metaphase II oocytes, *Cho* cholesterol, *CE* cholesteryl ester. **B** The GVBD rate of oocytes in the CTL (*n* = 128) and avasimibe (*n* = 146) groups. *GVBD* germinal vesicle breakdown. **C** Representative micrographs of oocytes after 14 h culture in the CTL and avasimibe groups. **D** The PBE rate of oocytes in the CTL (*n* = 128) and avasimibe (*n* = 146) groups. *PBE* polar body extrusion. **E** Representative images of spindle morphologies and chromosome alignment of MIIOs in the CTL and avasimibe groups. Scale bar, 25 μm. **F** The meiotic defects rate of MIIOs in the CTL (*n* = 51) and avasimibe (*n* = 44) groups. **G** Representative images of two-cell embryos from MIIOs in the CTL (*n* = 51) and avasimibe (*n* = 44) groups. CTL (control) group: MIIOs retrieved from MEMα maturation medium; avasimibe group: MIIOs retrieved from MEMα maturation medium supplemented with 20 μM avasimibe. **H** The two-cell embryos rate in the CTL (*n* = 51) and avasimibe (*n* = 44) groups. * *P* < 0.05; ** *P* < 0.01; **** *P* < 0.0001; *ns* not significant

To further explore the effect of cholesterol–cholesteryl ester metabolic homeostasis on oocytes, a pharmacological ACAT1 inhibitor, avasimibe, was used to inhibit the conversion of cholesterol to cholesteryl ester. The denuded GOs were treated with or without avasimibe for 14 h, and then the MIIOs were obtained to evaluate the validity of the inhibitor. Compared with the control group, avasimibe treatment considerably reduced cholesteryl ester levels and promoted free cholesterol accumulation (Fig. 3A). Consistently, the cholesteryl ester conversion rate was remarkably decreased in the avasimibe group than in the control group (Fig. 3A). After confirming the validity, we investigated the influence of avasimibe on oocyte meiosis. The results showed that 20 μM avasimibe treatment significantly decreased the PBE rate of oocytes, though it did not affect the GVBD rate (Fig. 3B–D). We next evaluated the spindle structure of MIIOs and observed typical barrel-like spindles with aligned chromosomes in most MIIOs in the control group, while the rate of meiotic defects was significantly higher in avasimibe-treated MIIOs (Fig. 3E, F). We further performed IVF to compare the early embryonic development of MIIOs from two groups. The result showed that, despite the low efficiency of conventional IVF in denuded oocytes, avasimibe treatment significantly reduced the two-cell embryo rate (Fig. 3G, H).

**Fig. 4:**
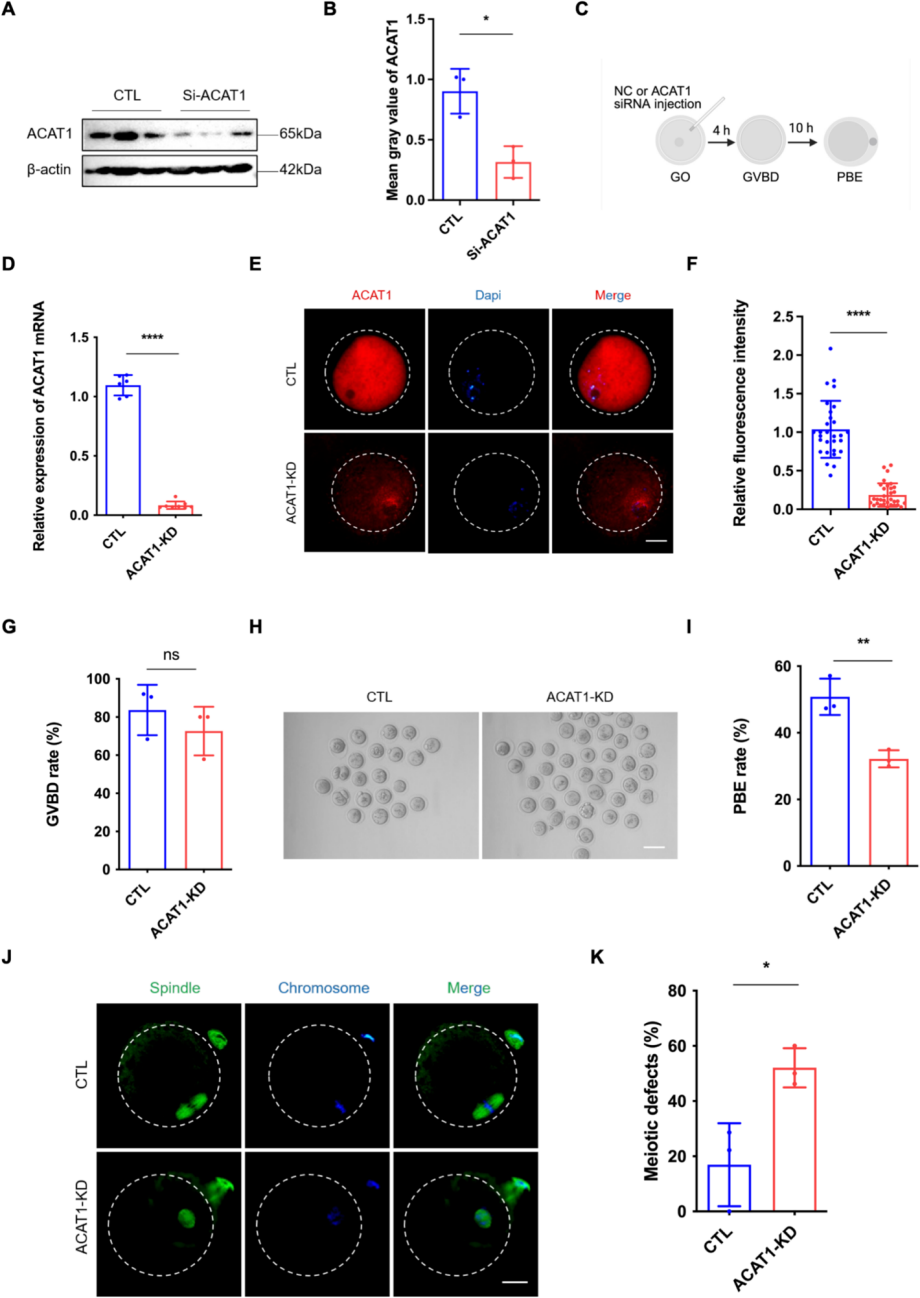
Disturbance of cholesterol–cholesteryl ester homeostasis by ACAT1 siRNA microinjection interferes with oocyte meiosis. **A** Protein expression of ACAT1 in the CTL and Si-ACAT1 groups. CTL (control) group: mouse Neuro-2a cells transfected with negative control siRNA; Si-ACAT1 group: mouse Neuro-2a cells transfected with ACAT1 siRNA. **B** Mean gray value of ACAT1 protein expression in the CTL and Si-ACAT1 groups. **C** Schematic diagram of oocyte microinjection and IVM. *NC* negative control, *GO* germinal vesicle oocyte, *GVBD* germinal vesicle breakdown, *PBE* polar body extrusion. **D** ACAT1 mRNA levels of oocytes in the CTL (*n* = 6) and ACAT1-KD (*n* = 8) groups. CTL (control) group: GO microinjection with negative control siRNA; ACAT1-KD group: GO microinjection with ACAT1 siRNA. **E** Representative images of oocyte immunofluorescence stained with ACAT1 in the CTL and ACAT1-KD groups. Scale bar, 25 μm.** F** Relative fluorescence intensity of oocyte immunofluorescence in the CTL (*n* = 29) and ACAT1-KD (*n* = 32) groups. **G** The GVBD rate of oocytes in the CTL (*n* = 130) and ACAT1-KD (*n* = 143) groups. **H** Representative micrographs of oocytes after 14 h culture in the CTL and ACAT1-KD groups. White arrowheads denote oocytes that failed to extrude a polar body. Scale bar, 100 μm. **I** The PBE rate of oocytes in the CTL (*n* = 130) and ACAT1-KD (*n* = 143) groups. **J** Representative images of spindle morphologies and chromosome alignment of MIIOs in the CTL and ACAT1-KD groups. MIIOs, metaphase II oocytes. Scale bar, 25 μm. **K** The meiotic defects rate of MIIOs in the CTL (*n* = 35) and ACAT1-KD (*n* = 48) groups. * *P* < 0.05; ** *P* < 0.01; **** *P* < 0.0001; *ns* not significant

To further validate the above findings, we used ACAT1 siRNA to inhibit ACAT1 expression. Western blotting results showed that ACAT1 siRNA significantly reduced the expression of ACAT1 protein in the mouse Neuro-2a cells (Fig. 4A, B). Next, the denuded GOs were microinjected with the siRNA to knock down the ACAT1 level (Fig. 4C). Immunofluorescence and single-oocyte qRT-PCR showed a significant knockdown efficiency of ACAT1 by microinjection (Fig. 4D–F). Consistent with the above results, ACAT1 knockdown significantly reduced the PBE rate and increased the meiotic defects rate, along with no observed difference in GVBD rate compared with the negative control group (Fig. 4G–K). Collectively, these findings demonstrate that disrupted cholesterol–cholesteryl ester metabolism interferes with oocyte quality.

### Impaired cholesterol–cholesteryl ester homeostasis affects oocyte quality through mitophagy

**Fig. 5:**
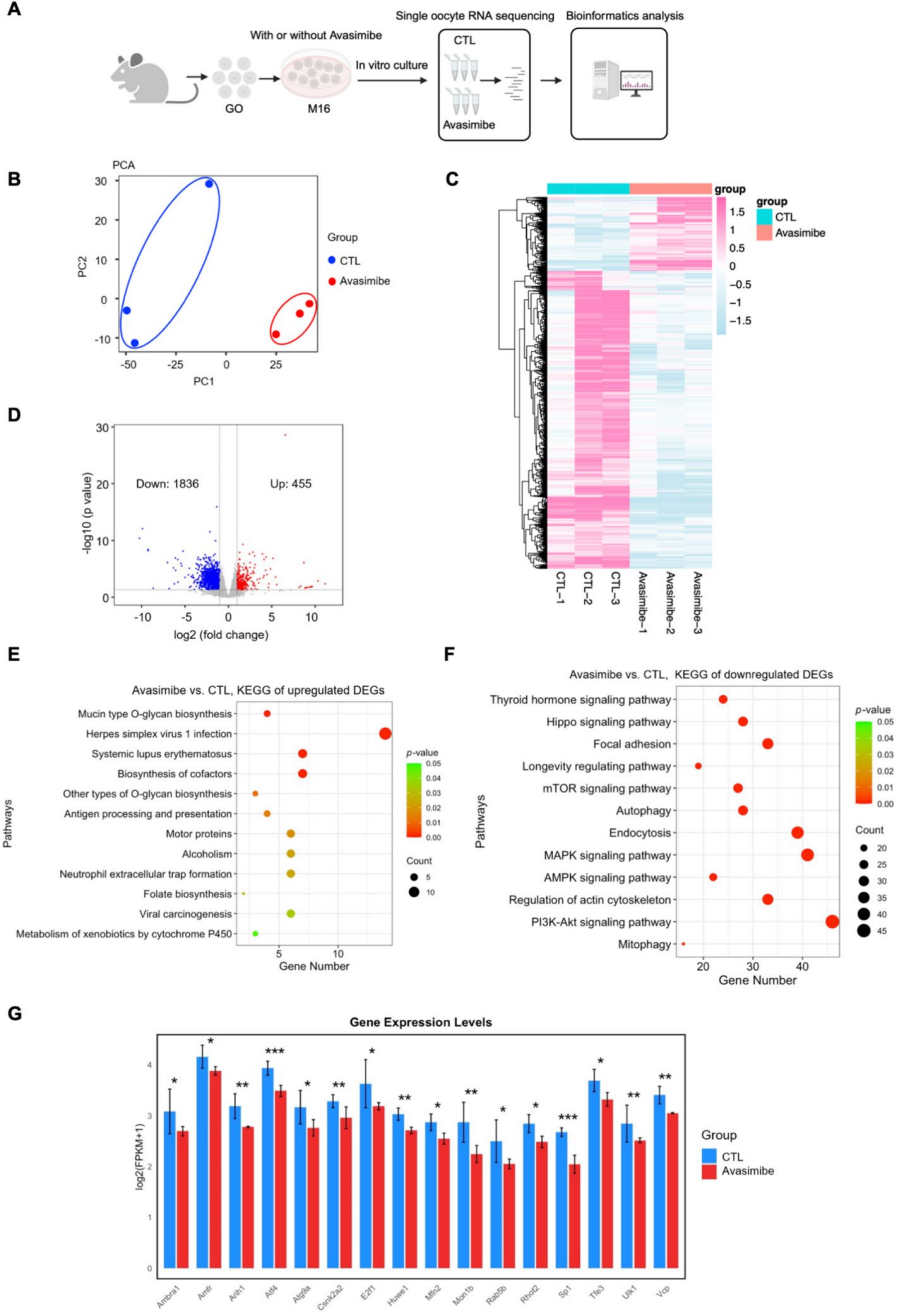
Single-oocyte transcriptomic analysis reveals the regulatory mechanism of impaired cholesterol–cholesteryl ester homeostasis effects on oocytes. **A** Overview of single-oocyte transcriptomic workflow. CTL (control) group: MIIOs obtained from M16 medium; avasimibe group: MIIOs obtained from M16 medium supplemented with 20 μM avasimibe. *MIIOs* metaphase II oocytes. *GO* germinal vesicle oocyte. **B** PCA of the RNA sequencing in the CTL and avasimibe groups. *PCA* principal component analysis. **C** Heatmap showing the DEGs in the CTL and avasimibe groups. *DEGs* differentially expressed genes.** D** Volcano plot showing the number of DEGs in the CTL and avasimibe groups. **E** KEGG enrichment analysis of upregulated DEGs. *KEGG* Kyoto Encyclopedia of Genes and Genomes. **F** KEGG enrichment analysis of downregulated DEGs. **G** The mRNA expression levels of mitophagy-associated genes in the CTL and avasimibe groups. * *P* < 0.05; ** *P* < 0.01; *** *P* < 0.001

To gain insight into the potential mechanism by which impaired cholesterol–cholesteryl ester homeostasis disturbs oocyte quality, single-oocyte transcriptomic and bioinformatics analyses were carried out. The flowchart is shown in Fig. 5A. In brief, denuded GOs were treated with or without avasimibe for 14 h, and then the MIIOs were collected for subsequent transcriptomic analysis. PCA could clearly distinguish the samples from the control and avasimibe-treated oocytes, revealing a significant genotype distinction between the two groups (Fig. 5B). Heatmap analysis showed a remarkably different oocyte transcriptomic profiling between the two groups (Fig. 5C). Data from volcano plots analysis revealed a total of 455 upregulated and 1836 downregulated DEGs in the avasimibe group compared with the control group (Fig. 5D). KEGG enrichment analysis showed that upregulated DEGs were related to mucin-type *O*-glycan biosynthesis, biosynthesis of cofactors, and other types of *O*-glycan biosynthesis (Fig. 5E). Interestingly, downregulated DEGs were mainly enriched in the phosphatidylinositol 3-kinase (PI3K)-Akt, mitogen-activated protein kinase (MAPK), mammalian target of rapamycin (mTOR), and Hippo signaling pathways (Fig. 5F), which were involved in multiple aspects of oocyte growth, maturation, and development [32–34]. Moreover, these downregulated DEGs were also enriched in the endocytosis, autophagy, and mitophagy processes (Fig. 5F). The expression of the mitophagy-associated genes, including *Amfr*, *Atf4*, *Tfe3*, *Atg9a*, *Ambra1*, *Arih1*, *Csnk2a2*, *E2f1*, *Huwe1*, *Mfn2*, *Mon1b*, *Rab5b*, *Rhot2*, *Sp1*, *Ulk1*, and *Vcp*, was significantly downregulated in the avasimibe-treated group (Fig. 5G).These results suggest that the oocyte abnormalities induced by impaired cholesterol–cholesteryl ester homeostasis are mediated through mitophagy.

**Fig. 6:**
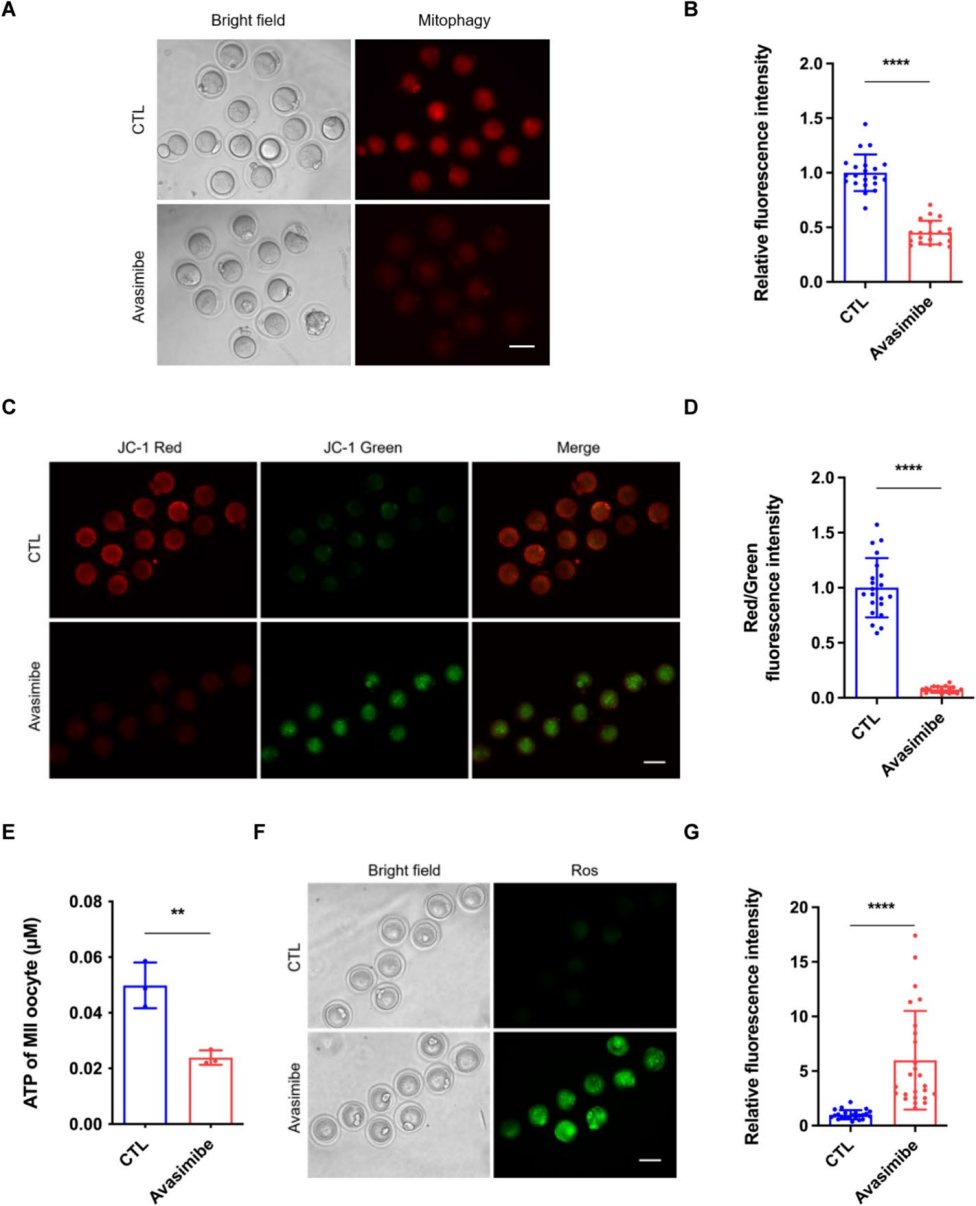
Inhibition of cholesterol conversion to cholesteryl ester impairs oocyte mitophagy and mitochondrial function. **A** Representative micrographs of mitophagy staining of MIIOs in the CTL and avasimibe groups. CTL (control) group: oocytes cultured in M16 medium; avasimibe group: oocytes cultured in M16 medium supplemented with 20 μM avasimibe. MIIOs, metaphase II oocytes. Scale bar, 100 μm. **B** Relative fluorescence intensity of mitophagy staining in the CTL (*n* = 21) and avasimibe (*n* = 20) groups. **C** Representative micrographs of JC-1 staining of MIIOs in the CTL and avasimibe groups. Scale bar, 100 μm. **D** Relative fluorescence intensity of JC-1 staining in the CTL (*n* = 21) and avasimibe (*n* = 19) groups. **E** Quantification of ATP in individual MII oocyte in the CTL (*n* = 30) and avasimibe (*n* = 30) groups. **F** Representative micrographs of ROS staining of MIIOs in the CTL and avasimibe groups. *ROS* reactive oxygen species. Scale bar, 100 μm.** G** Relative fluorescence intensity of ROS staining in the CTL (*n* = 23) and avasimibe (*n* = 25) groups. ** *P* < 0.01; **** *P* < 0.0001

To confirm whether inhibiting cholesterol conversion to cholesteryl ester would damage mitophagy, we performed the mitophagy detection in MIIOs and found that avasimibe treatment significantly decreased the level of mitophagy (Fig. 6A, B). Considering the controlling effect of mitophagy on mitochondrial function, we further examined the mitochondrial function. Avasimibe treatment contributed to the loss of oocyte MMP (Fig. 6C and D). Further detection of ATP production showed considerably decreased ATP levels in oocytes treated with avasimibe (Fig. 6E). We also detected mitochondrial ROS production. We observed that avasimibe-treated oocytes showed excessive ROS production (Fig. 6F, G). Collectively, these findings demonstrate that impaired cholesterol–cholesteryl ester homeostasis affects oocyte quality through mitophagy.

### Cholesterol–cholesteryl ester metabolic homeostasis is imbalanced in aged oocytes

**Fig. 7:**
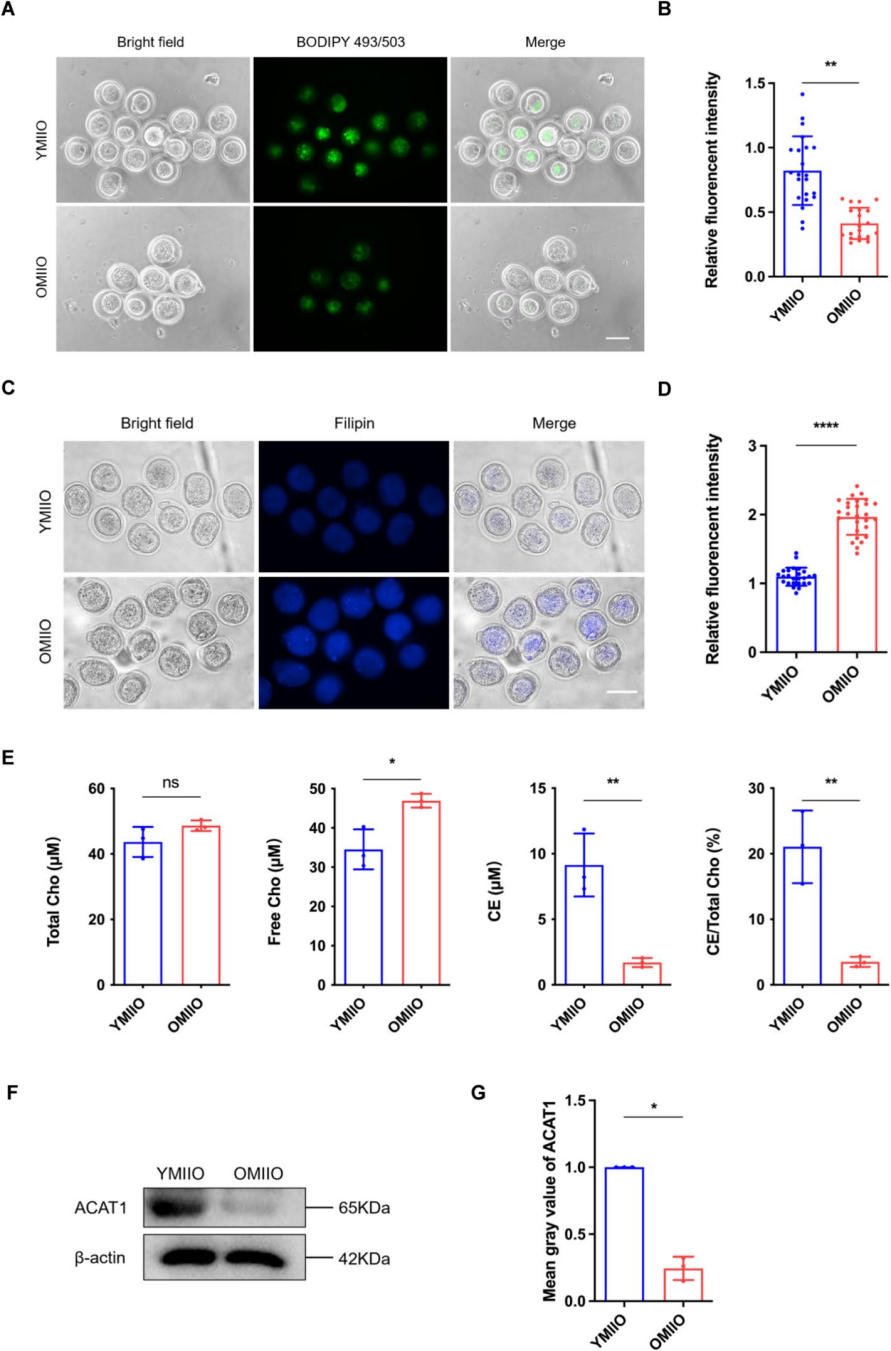
Homeostasis of cholesterol–cholesteryl ester metabolism is imbalanced in aged oocytes. **A** Representative images of BODIPY 493/503 staining in the YMIIO and OMIIO groups. YMIIO group: young metaphase II oocyte retrieved from M16 medium; OMIIO group: aged metaphase II oocyte retrieved from M16 medium. Scale bar, 100 μm.** B** Relative fluorescence intensity of BODIPY 493/503 staining in the YMIIO (*n* = 22) and OMIIO (*n* = 20) groups. **C** Representative images of Filipin staining in the YMIIO and OMIIO groups. Scale bar, 100 μm.** D** Relative fluorescence intensity of Filipin staining in the YMIIO (*n* = 25) and OMIIO (*n* = 27) groups. **E** Quantification of total cholesterol, free cholesterol, cholesteryl ester, and cholesteryl ester conversion rate in the YMIIO (*n* = 150) and OMIIO (*n* = 150) groups. *Cho* cholesterol, *CE* cholesteryl ester. **F** Protein expression of ACAT1 in the YMIIO (*n* = 210) and OMIIO (*n* = 210) groups. **G** Mean gray value of ACAT1 protein expression in the YMIIO and OMIIO groups. * *P* < 0.05; ** *P* < 0.01; **** *P* < 0.0001; *ns* not significant

As our above findings suggest, the homeostasis of cholesterol–cholesteryl ester metabolism plays a crucial role in oocyte quality, but its contribution to oocyte aging remains elusive. To investigate whether impaired cholesteryl ester conversion was involved in the disruption of oocyte quality during ovarian aging, we first examined the cholesteryl ester and free cholesterol levels in MIIOs from young (6-week-old) and aged (10-month-old) mice. BODIPY 493/503 staining showed significantly decreased cholesteryl ester levels in the aged compared with the young MIIOs (Fig. 7A, B). By contrast, Filipin staining showed higher free cholesterol accumulation in the aged MIIOs (Fig. 7C, D). Consistently, quantification detection of cholesterol-related metabolites also revealed that, compared with the young MIIOs, the aged MIIOs had lower cholesteryl ester levels and cholesteryl ester conversion rates along with higher free cholesterol (Fig. 7E). In addition, we also investigated the ACAT1 expression, and the western blotting result showed significantly decreased ACAT1 levels in aged compared with young MIIOs (Fig. 7F, G). Together, these results demonstrate that the homeostasis of cholesterol–cholesteryl ester metabolism is impaired, accompanied by reduced ACAT1 expression, in aged oocytes.

### Amelioration of cholesterol metabolic homeostasis improves aged oocyte quality via mitophagy

**Fig. 8:**
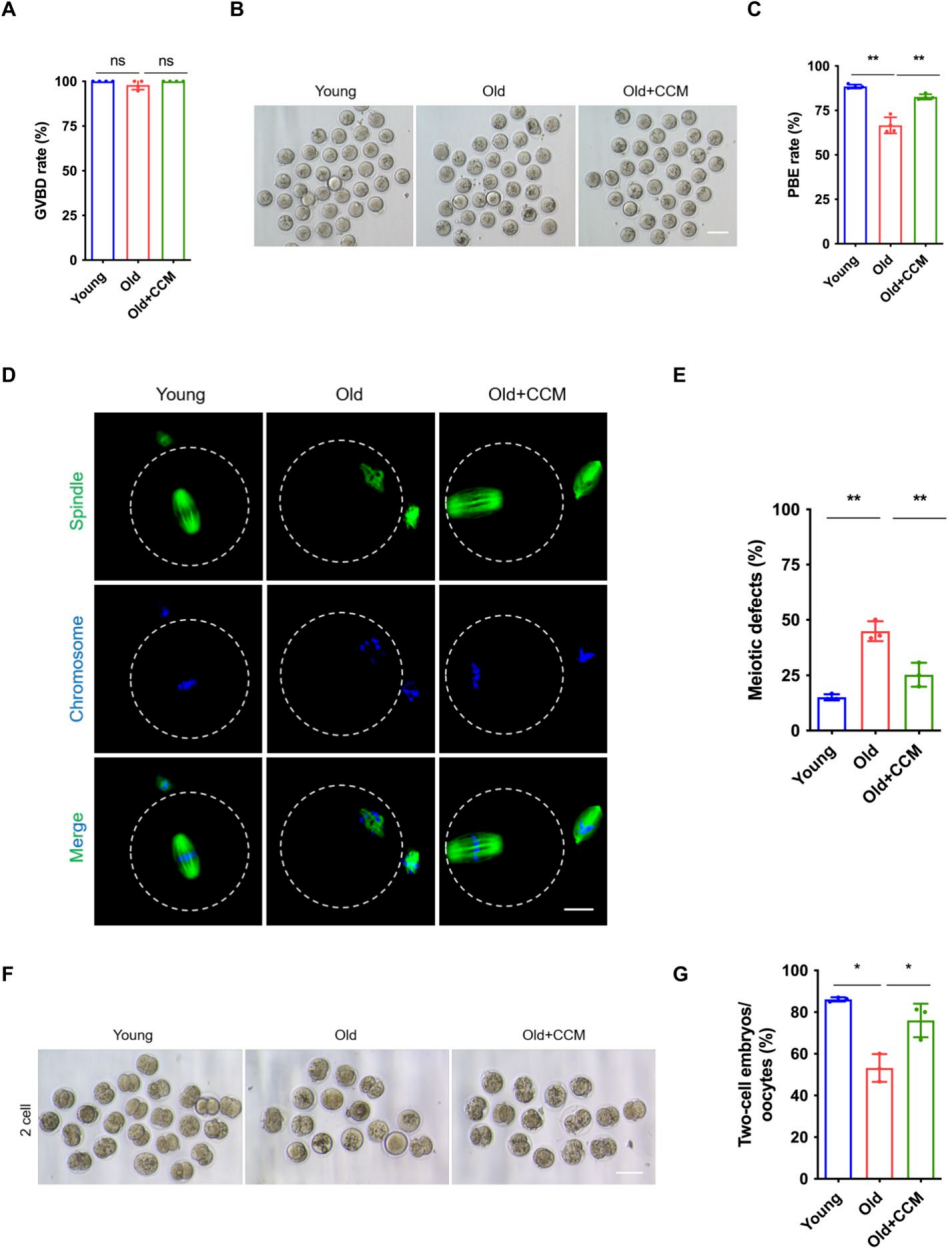
Amelioration of cholesterol metabolic homeostasis improves aged oocyte quality. **A** The GVBD rate of oocytes in the young (*n* = 90), old (*n* = 72), and old + CCM (*n* = 67) groups. Young group: young COCs cultured in M16 medium; old group: aged COCs cultured in M16 medium; old + CCM group: aged COCs cultured in M16 medium supplemented with 20 μM CCM. *COCs* cumulus–oocyte complexes, *CCM* cholesterol conjugated to methyl-β-cyclodextrin, *GVBD* germinal vesicle breakdown. **B** Representative micrographs of oocytes after 14 h culture in the young, old, and old + CCM groups. Scale bar, 100 μm. **C** The PBE rate of oocytes in the young (*n* = 90), old (*n* = 72), and old + CCM (*n* = 67) groups. *PBE* polar body extrusion. **D** Representative images of spindle morphologies and chromosome alignment of MIIOs in the young, old, and old + CCM groups. Scale bar, 25 μm. *MIIOs* metaphase II oocytes. **E** The meiotic defects rate of MIIOs in the young (*n* = 45), old (*n* = 33), and old + CCM (*n* = 37) groups. **F** Representative images of two-cell embryos from MIIOs in the young, old, and old + CCM groups. Young group, young COCs cultured in MEMα maturation medium; old group: aged COCs cultured in MEMα maturation medium; old + CCM group: aged COCs cultured in MEMα maturation medium supplemented with 20 μM CCM. **G** The two-cell embryos rate in the young (*n* = 108), old (*n* = 57), and old + CCM (*n* = 60) groups. * *P* < 0.05; ** *P* < 0.01; *ns* not significant

To explore whether ameliorating cholesterol–cholesteryl ester metabolism abnormalities would improve aged oocyte quality, we performed a rescue assay. Cholesteryl ester-rich conditions were achieved by CCM treatment according to a previous report [30]. Considering that the process of oocyte cholesterol uptake depends on support from the surrounding granulosa cells, we added the CCM to the COCs for IVM. Our results showed that CCM treatment significantly increased the PBE rates of aged oocytes similar to young oocytes, though with no difference in the GVBD rate compared with the control group (Fig. 8A–C). Furthermore, spindle-chromosome structure examination showed that CCM treatment rescued irregularly assembled spindles with misaligned chromosomes and significantly decreased meiotic defect rates in MIIOs from aged mice (Fig. 8D and E). The IVF result showed that CCM treatment effectively improved the two-cell embryo rate in the older group, being comparable to that in the young group (Fig. 8F, G).

**Fig. 9:**
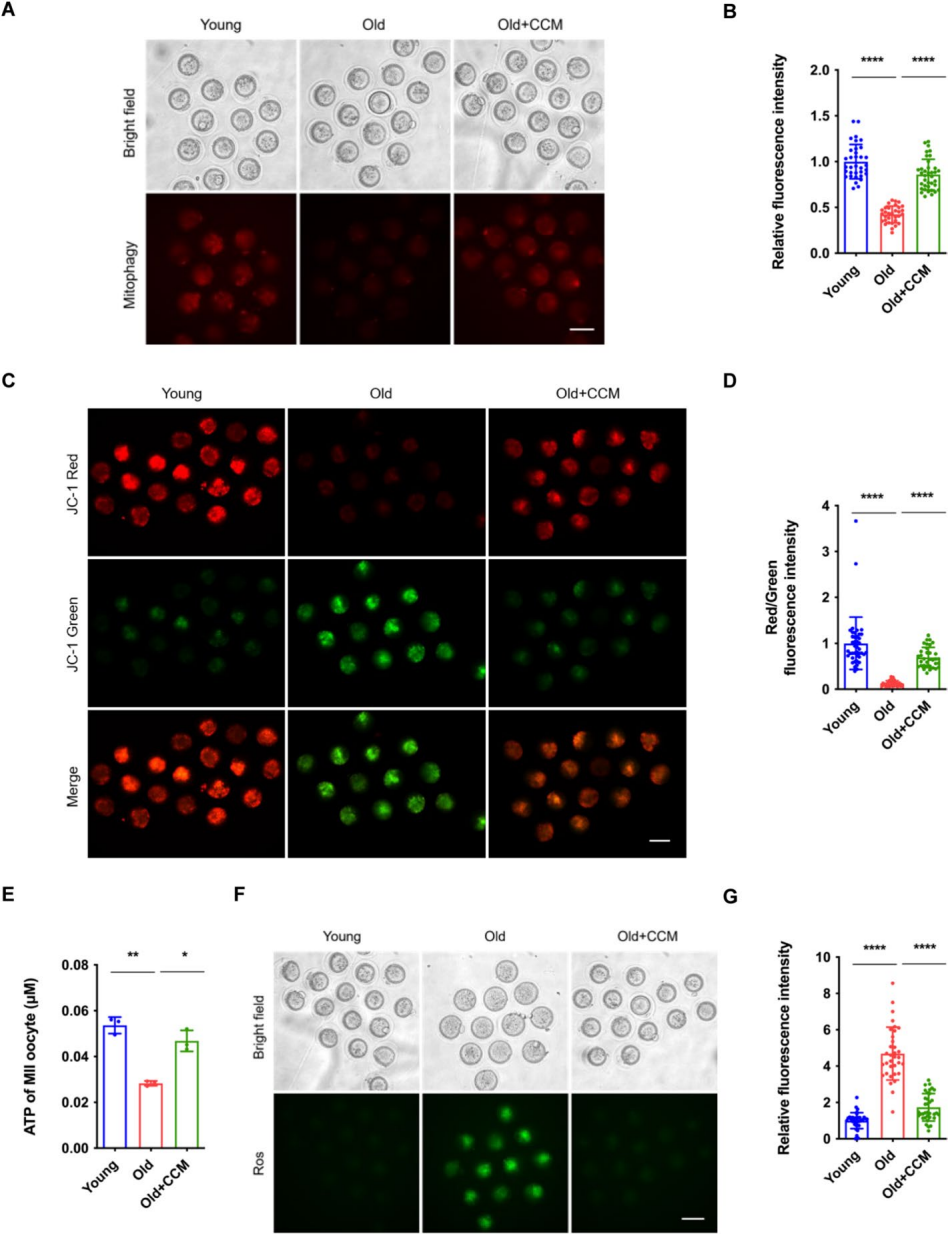
Amelioration of cholesterol metabolic homeostasis improves aged oocyte mitophagy and mitochondrial function. **A** Representative images of mitophagy staining of MIIOs in the young, old, and old + CCM groups. Young group: young COCs cultured in M16 medium; old group: aged COCs cultured in M16 medium; old + CCM group: aged COCs cultured in M16 medium supplemented with 20 μM CCM. *COCs* cumulus-oocyte complexes, *CCM* cholesterol conjugated to methyl-β-cyclodextrin. *MIIOs* metaphase II oocyte. Scale bar, 100 μm. **B** Relative fluorescence intensity of mitophagy staining in the young (*n* = 34), old (*n* = 32), and old + CCM (*n* = 37) groups. **C** Representative micrographs of JC-1 staining of MIIOs in the young, old, and old + CCM groups. Scale bar, 100 μm.** D** Relative fluorescence intensity of JC-1 staining in the young (*n* = 42), old (*n* = 34), and old + CCM (*n* = 34) groups. **E** Quantification of ATP in individual MII oocyte in the young (*n* = 30), old (*n* = 30), and old + CCM (*n* = 30) groups.** F** Representative micrographs of ROS staining of MIIOs in the young, old, and old + CCM groups. *ROS* reactive oxygen species. Scale bar, 100 μm.** G** Relative fluorescence intensity of ROS staining in the young (*n* = 36), old (*n* = 34), and old + CCM (*n* = 35) groups. * *P* < 0.05; ** *P* < 0.01; **** *P* < 0.0001

We next evaluated the effect of CCM on mitophagy and mitochondrial function in aged MIIOs. As expected, CCM treatment also significantly improved the mitophagy of aged oocytes, being comparable to that of young oocytes (Fig. 9A and B). Consistently, the detection of MMP and ATP content in aged oocytes confirmed that CCM supplementation remarkably recovered mitochondrial MMP and ATP production (Fig. 9C–E). In addition, CCM supplementation also effectively reduced the levels of ROS (Fig. 9F, G). In summary, these results demonstrate that ameliorating cholesterol–cholesteryl ester metabolic homeostasis recovers aged oocyte quality via mitophagy.

## Discussion

Owing to delayed childbearing and increase in the childbearing age of women, the decline of oocyte quality as ovarian aging has become a major social concern [35, 36]. A lot of studies have shown that age-related metabolic changes may affect oocyte quality [37–39]. However, to date, our understanding of the intrinsic regulation of oocyte quality by metabolites, metabolic enzymes, and intracellular mediators is less well-characterized. In the current study, we identified that oocytes underwent a dramatic increase in cholesterol conversion to cholesteryl ester during meiotic maturation. Furthermore, we demonstrated that impaired transformation of cholesterol to cholesteryl ester interfered with oocyte quality and participated in oocyte aging. These findings together provide a novel insight into the vital role of homeostasis of cholesterol–cholesteryl ester metabolism in oocyte quality.

Cholesterol exists in two forms: free (unesterified) cholesterol and cholesteryl ester (esterified cholesterol). The former is an active form present in the cell membrane, whereas the latter is stored in LDs. Homeostasis of cholesterol metabolism is crucial for oocytes. According to previous reports, cholesterol depletion in the plasma membrane of oocytes in mice can reduce the success rate of IVF [25]. In addition, excess cholesterol can also trick mouse oocytes into behaving as though they were fertilized, thus disrupting the normal synchrony between fertilization and completion of meiosis [26]. Excessive free cholesterol in cells is reported to induce cytotoxicity [40]. Currently, our work has found a drastic increase in cholesterol conversion to cholesteryl ester during normal oocyte meiotic maturation. This conversion depends on ACAT1, which can regulate cholesterol esterification and prevent the accumulation of free cholesterol, maintaining the homeostasis of cholesterol–cholesteryl ester metabolism in oocytes. We also confirmed that impaired transformation of cholesterol to cholesteryl ester significantly interfered with oocyte quality and early embryonic development, which could be shown to result from the unnecessary accumulation of free cholesterol.

Mitochondria act as a central hub that integrates many cellular processes, such as metabolic energy production, cellular redox balance, as well as ROS homeostasis [41, 42]. Mitophagy is a process of mitochondrial quality control for removing damaged or defective mitochondria and is considered to be an “initiator” in follicular abnormal atresia, meiosis, and delayed embryonic development [43–46]. Therefore, mitophagy is crucial to maintain oocyte mitochondrial function and quality. It has been reported that accumulation of cholesterol can induce abnormal mitochondrial function, including the limitation of crucial antioxidant defenses, increased generation of ROS, and defective assembly of respiratory supercomplexes [47, 48]. Impaired mitophagy has also been linked to the accumulation of intracellular cholesterol [49, 50], which aligns with our current results. We confirmed that impaired transformation of cholesterol to cholesteryl ester in oocytes impaired mitophagy and further disrupted mitochondrial function, including ATP generation, MMP loss, and ROS overload, which finally led to oocyte abnormal meiotic maturation and quality. It is generally accepted that mitochondrial dysfunction is one of the hallmarks of ovarian aging [15, 51, 52]. Recent findings also highlighted the potential role of targeting mitophagy in improving aged oocyte quality [13, 53]. In this study, we further detected impaired homeostasis of cholesterol–cholesteryl ester metabolism in aged oocytes, primarily manifested as reduced cholesteryl ester and increased free cholesterol levels. Considering the potential regulatory role of cholesterol–cholesteryl ester metabolism in mitochondria, we thus conducted cholesteryl ester supplementation and demonstrated that it can effectively restore aged oocyte quality via improving mitophagy and mitochondrial function. These findings together suggest the mechanism by which cholesterol–cholesteryl ester metabolism regulates oocyte quality and thus participates in the process of ovarian aging by influencing mitochondrial autophagy and mitochondrial function.

Both cholesteryl ester and TAG constitute the core content of LDs. A previous study reported that bovine oocytes accumulated TAG during IVM [54]. Consistently, our current results showed that the cholesteryl ester levels were increased with oocyte maturation, which suggested LD accumulation during oocyte meiotic maturation. More and more studies have emphasized LDs as critical organelles in lipid metabolism, energy regulation, and efficient mitophagy [55, 56]. Currently, our results show that impaired cholesterol conversion to cholesteryl ester decreased cholesteryl esterification, which thus reduced the availability of LDs and damaged oocyte mitophagy and mitochondrial function. The physical and functional contacts between LDs and mitochondria can facilitate a direct transfer of lipids, which are crucial for mitochondrial β-oxidation, thus influencing energy homeostasis [57, 58]. In this study, we assumed that impaired homeostasis of cholesterol–cholesteryl ester metabolism decreased the availability of LDs, which thus may disturb mitophagy and mitochondrial function. Conversely, cholesteryl ester supplementation improved aged oocyte mitophagy and mitochondrial function, including enhancing ROS scavenging capacity, MMP, and ATP levels, thus increasing PBE and decreasing meiotic defect rates. A study based on glutathione deficiency also observed that reduced developmental competence in oocytes was accompanied by reduced LD storage and increased mitochondrial dysfunction [59]. However, we still do not know through which pathway LDs in oocytes interact with the mitochondria, which needs further study.

Some other limitations of this study should be noted. First, the oocytes and their surrounding granulosa cells are metabolically interdependent according to wide reports [60–62]. Considering that the oocyte cannot take up cholesterol independently, the mechanism by which the granulosa cells surrounding the oocyte communicate and transfer cholesterol-related metabolites to the oocyte remains elusive and is worthy of further study. Secondly, although the defects in aged oocytes do not occur on the basis of post-ovulation aging, more in vivo experiments deserve attention, which will be a subject for our future work. Finally, although these discoveries are based on a good mouse model of ovarian aging that has been validated in our previous report, there is still a species limitation. Future work focusing on human oocytes could provide more convincing evidence. Overall, our study provides a comprehensive understanding of cholesterol–cholesteryl ester metabolic homeostasis in regulating oocyte quality, which may provide a novel view of the potential implications of cholesterol-related metabolites in aged oocytes.

## Conclusions

We reveal that increased conversion of cholesterol to cholesteryl ester is necessary for maintaining oocyte quality. Conversely, impaired homeostasis of cholesterol–cholesteryl ester metabolism interferes with oocyte quality in the process of ovarian aging. Regarding the mechanism, inhibition of cholesteryl ester conversion damaged oocyte mitophagy, which thus led to mitochondrial dysfunction, including reduced mitochondrial membrane potential and ATP production, and excess accumulation of ROS.

## Supplementary Information

Supplementary Material 1.

## Data Availability

The data that support the findings of this study are available from the corresponding author as needed.

## Abbreviations

ACAT1/2: Acyl-coenzyme A: cholesterol acyltransferase 1/2
ATP: Adenosine triphosphate
BCA: Bicinchoninic acid
BODIPY: 493/503 4,4-difluoro-1,3,5,7,8-pentamethyl-4-bora-3a,4a-diaza-s-indacene
CCM: Cholesterol conjugated to methyl-β-cyclodextrin
cDNA: Complementary DNA
COC: Cumulus–oocyte complex
DAG: Diacylglycerol
DAPI: 4’,6-diamidino-2-phenylindole
DEG: Differentially expressed gene
DMEM: Dulbecco’s modified Eagle’s medium
GO: Germinal vesicle oocyte
GVBD: Germinal vesicle breakdown
HCG: Human chorionic gonadotropin
HPLC: High-performance liquid chromatography
HRP: Horseradish peroxidase
IVF: In vitro fertilization
IVM: In vitro maturation
KEGG: Kyoto Encyclopedia of Genes and Genomes
KSOM: Potassium simplex optimized medium
LD: Lipid droplet
MAPK: Mitogen-activated protein kinase
MIIO: Metaphase II oocyte
MMP: Mitochondrial membrane potential
mTOR: Mammalian target of rapamycin
PAGE: Polyacrylamide gel electrophoresis
PBE: Polar body extrusion
PBS: Phosphate-buffered saline
PBST: Phosphate-buffered saline with Tween-20
PCA: Principal component analysis
PI3K: Phosphatidylinositol 3-kinase
PMSG: Pregnant mare serum gonadotropin
qRT-PCR: Quantitative real-time polymerase chain reaction
ROS: Reactive oxygen species
SDS: Sodium dodecyl sulfate
siRNA: Small interfering RNA
TAG: Triacylglycerol
