# Supplementary material for: Abnormal cholesterol–cholesteryl ester metabolism impairs mouse oocyte quality during ovarian aging
Source: Cell Mol Biol Lett. 2025 Nov 24;30:140. doi: 10.1186/s11658-025-00811-w (PMC12642260; doi:10.1186/s11658-025-00811-w)

Marker Thermo Scientific™ 26617

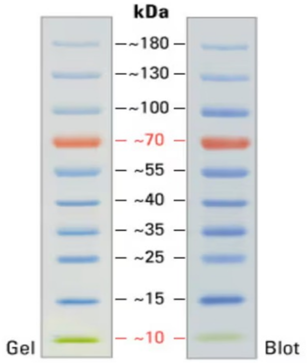

ACAT1 GO MIIO-1

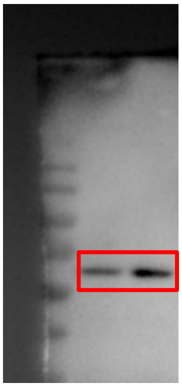

ACAT2 GO MIIO-1

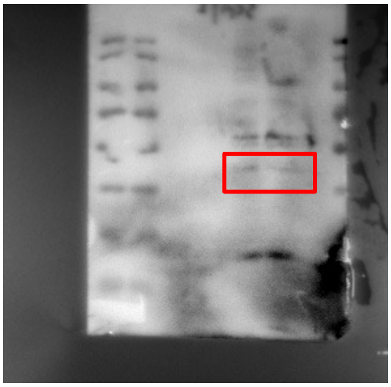

β-actin GO MIIO-1

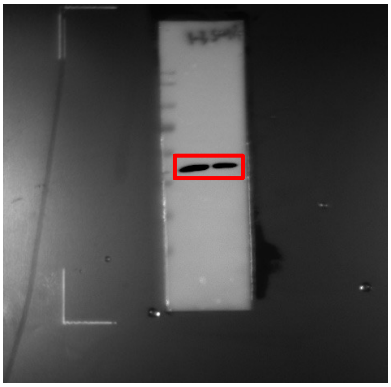

ACAT1 GO MIIO-2

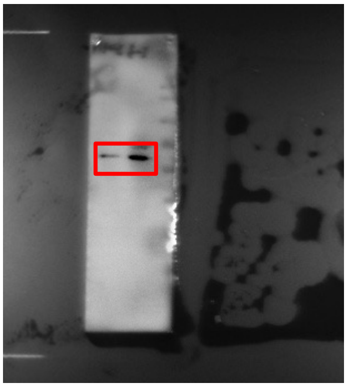

ACAT2 GO MIIO-2

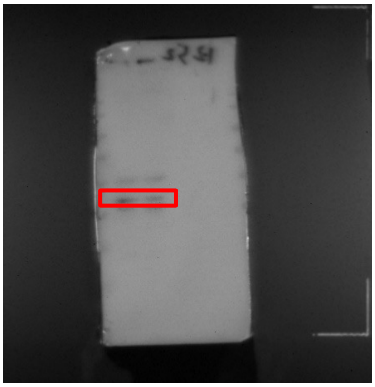

β-actin GO MIIO-2

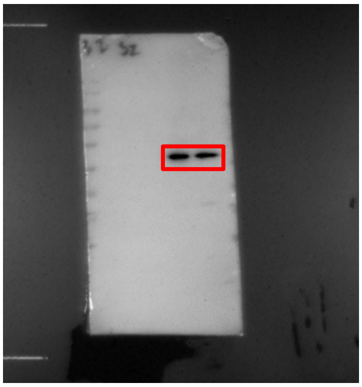

ACAT1 GO MIIO-3

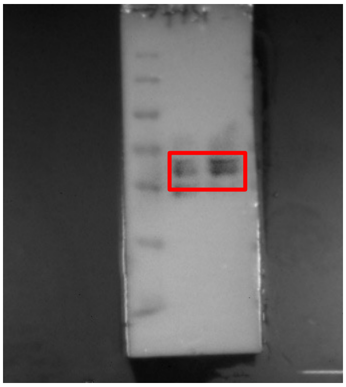

ACAT2 GO MIIO-3

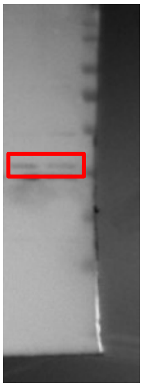

β-actin GO MIIO-3

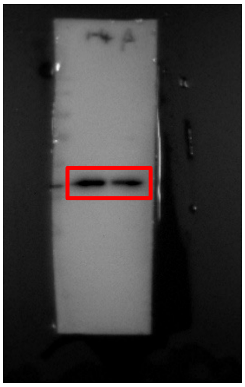

ACAT1 Ctl si-1

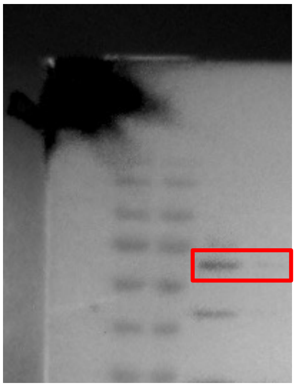

β-actin Ctl si-1

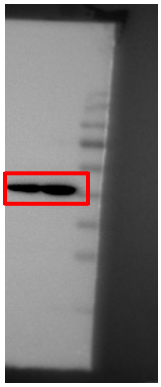

ACAT1 Ctl1,2,3 si1,2,3 -2

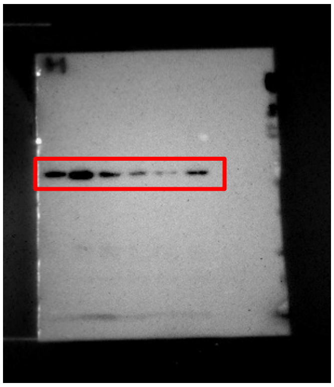

β-actin Ctl1,2,3 si1,2,3 -2

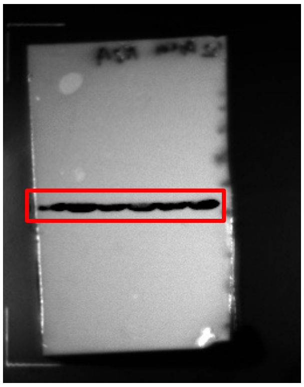

ACAT1 Ctl si-3

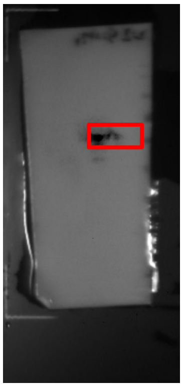

β-actin Ctl si-3

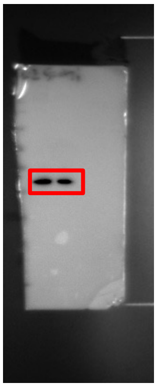

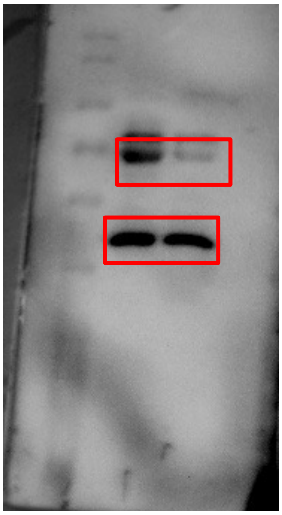

ACAT1
YMIIO-1 OMIIO-1

β-actin
YMIIO-1 OMIIO-1

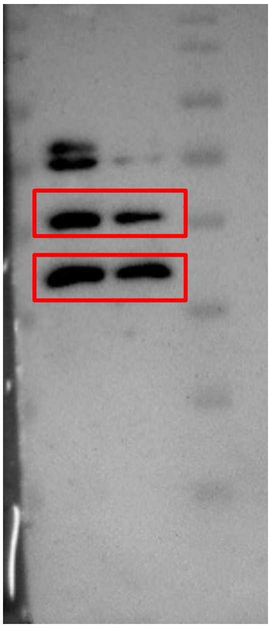

ACAT1
YMIIO-2 OMIIO-2

β-actin
YMIIO-2 OMIIO-2

ACAT1
YMIIO-3 OMIIO-3

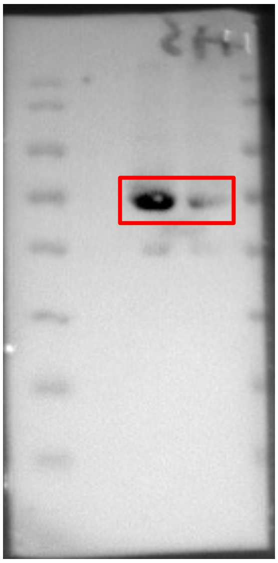

β-actin
YMIIO-3 OMIIO-3

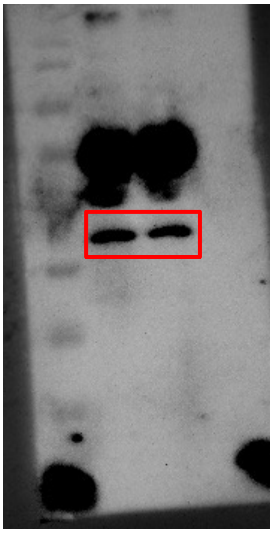

Supplement: Supplementary file 1 — Supplementary Material 1. [file 11658_2025_811_MOESM1_ESM.pdf]
